# Pasireotide does not improve efficacy of aspiration sclerotherapy in patients with large hepatic cysts, a randomized controlled trial

**DOI:** 10.1007/s00330-017-5205-1

**Published:** 2018-01-09

**Authors:** Titus F. M. Wijnands, Tom J. G. Gevers, Marten A. Lantinga, René H. te Morsche, Leo J. Schultze Kool, Joost P. H. Drenth

**Affiliations:** 1 Department of Gastroenterology and Hepatology, Radboud University Medical Centre, P.O. Box 9101, code 455, 6500 HB Nijmegen, The Netherlands; 2 Department of Radiology, Radboud University Medical Centre, Nijmegen, The Netherlands

**Keywords:** Liver, Cyst, Sclerotherapy, Somatostatin, Randomized controlled trial

## Abstract

**Objectives:**

We tested whether complementary use of the somatostatin analogue pasireotide would augment efficacy of aspiration sclerotherapy of hepatic cysts.

**Methods:**

We conducted a double-blind, placebo-controlled trial in patients who underwent aspiration sclerotherapy of a large (>5 cm) symptomatic hepatic cyst. Patients were randomized to either intramuscular injections of pasireotide 60 mg long-acting release (n = 17) or placebo (sodium chloride 0.9 %, n = 17). Injections were administered 2 weeks before and 2 weeks after aspiration sclerotherapy. The primary endpoint was proportional cyst diameter reduction (%) from baseline to 6 weeks. Secondary outcomes included long-term cyst reduction at 26 weeks, patient-reported outcomes including the polycystic liver disease-questionnaire (PLD-Q) and safety.

**Results:**

Thirty-four patients (32 females; 53.6 ± 7.8 years) were randomized between pasireotide or placebo. Pasireotide did not improve efficacy of aspiration sclerotherapy at 6 weeks compared to controls (23.6 % [IQR 12.6–30.0] vs. 21.8 % [9.6–31.8]; *p* = 0.96). Long-term cyst diameter reduction was similar in both groups (49.1 % [27.0–73.6] and 45.6 % [29.6–59.6]; *p* = 0.90). Mean PLD-Q scores improved significantly in both groups (*p* < 0.01) without differences between arms (*p* = 0.92).

**Conclusions:**

In patients with large symptomatic hepatic cysts, complementary pasireotide to aspiration sclerotherapy did not improve cyst reduction or clinical response.

**Key Points:**

• *Complementary pasireotide treatment does not improve efficacy of aspiration sclerotherapy*.

• *Cyst fluid reaccumulation after aspiration sclerotherapy is a transient phenomenon.*

• *Aspiration sclerotherapy strongly reduces symptoms and normalizes quality of life.*

**Electronic supplementary material:**

The online version of this article (10.1007/s00330-017-5205-1) contains supplementary material, which is available to authorized users.

## Introduction

Hepatic cysts are fluid-filled cavities in the liver that congenitally arise from aberrant cholangiocytes [1]. Cysts occur in a spectrum from sporadic lesions to numerous cysts as part of polycystic liver disease (PLD). Due to continuous epithelial cell proliferation and fluid production, cysts grow over time [2, 3]. Large hepatic cysts lead to invalidating symptoms as pain, nausea and dyspnoea due to mechanical compression of surrounding organs that ultimately compromise the patients’ quality of life [4–6].

Aspiration sclerotherapy is a minimally invasive procedure aimed at reducing the volume of these fluid-filled lesions [7]. Injecting ethanol (or other sclerosing agents) directly in the cyst cavity destroys the inner cyst-lining epithelium [8–10]. Lysis of these epithelial cells halts cell proliferation and cyst fluid production [11]. Aspiration sclerotherapy generally reaches favourable results in solitary cysts or dominant cysts in PLD [7]. However, reported long-term efficacy of cyst reduction is mixed and ranges from complete regression to recurrence of the cyst [12, 13]. An important predictor of cyst reduction is the extent of intracystic fluid reaccumulation after treatment [14]. Typically, cyst fluid reaccumulates in the first weeks after treatment [15]. It is hypothesized that this transient fluid production may result from remnants of the cyst wall that are not eliminated by sclerotherapy [16]. This initial fluid relapse threatens long-term efficacy and may lead to recurrence of the cyst necessitating re-intervention [14, 15].

Somatostatin analogues inhibit cyst fluid production and cell proliferation by activating somatostatin receptors on the basolateral surface of the cyst [3, 17]. Previous studies showed that lanreotide and octreotide reduce cystic volume in patients with polycystic liver disease [18, 19]. Pasireotide (SOM230) is a more potent somatostatin analogue due to a broader binding profile and higher affinity to somatostatin receptor subtypes [20].

Adjuvant treatment of pasireotide with aspiration sclerotherapy has the promise of curtailing fluid reaccumulation and reducing cholangiocyte proliferation. This strategy has precedence as surgeons have described the use of octreotide after surgical cyst fenestration in an observational study (n = 5) and observed minimized fluid production by exposed remnants of fenestrated cysts leading to apparent control of ascites [21].

We hypothesized that the combined approach of aspiration sclerotherapy and pasireotide would decrease fluid accumulation and improve long-term cyst reduction and clinical response. Therefore, the purpose of this study was to test whether pasireotide could improve the efficacy of aspiration sclerotherapy of large symptomatic hepatic cysts.

## Materials and methods

This study was performed following approval by the Institutional Review Board (Medical Research Ethics Committee of the region Arnhem-Nijmegen) and conformed to the ethical guidelines of the 1975 Declaration of Helsinki. The trial was registered at ClinicalTrials.gov (NCT02048319). We previously reported a detailed protocol elsewhere [22]; a brief description is given below.

### Study design and participants

This randomized, single-centre, double-blind, placebo-controlled trial was carried out in the Radboud University Centre, Nijmegen, The Netherlands between April 2014 and April 2016. Trial duration was 26 weeks. Following written informed consent, we randomized patients in a 1:1 ratio to pasireotide or placebo. Randomization was concealed to patients and investigators.

Patients aged between 18 and 70 years with a large (> 5 cm) symptomatic solitary or dominant hepatic cyst were eligible for participation. A dominant cyst was regarded as the largest cyst within PLD. Major exclusion criteria were: a complicated cyst (neoplastic or hydatid cyst), a prolonged corrected QT-interval (> 470 ms), symptomatic cholecystolithiasis, uncontrolled diabetes (HbA1C > 64 mmol/mol) and surgical or radiological cyst interventions within 6 months prior to the study.

### Study procedures

All patients received aspiration sclerotherapy. In brief, we performed a single-session sclerotherapy procedure. The procedure was performed using conscious sedation with propofol (propofol 10 mg/ml, Fresenius, Bad Homburg vor der Höhe, Germany) and alfentanyl (rapifen, 5 mg/10 ml, Tilburg, The Netherlands). Guided by ultrasound, the interventional radiologist inserted a 5 French pigtail catheter (Cook Medical, Bloomington, IL, USA) in the cyst and performed fluid drainage until collapse of the cyst. Cyst leakage was ruled out by instillation of contrast medium (Iomeron 300, Bracco Imaging, Konstanz, Germany, up to 20 ml) followed by 10-min sclerotherapy. We injected 100 % ethanol in a 10 % ratio of the aspirated cyst volume (up to 50 ml) [23]. All patients were hospitalized for 24-h observation.

The intervention arm received 60 mg pasireotide (SOM230, Novartis, Basel, Switzerland) long-acting release (LAR). This formulation is injected intramuscularly and secures an extended-release depot suitable for monthly administration [20]. Based on its pharmacokinetic profile we administered pasireotide 2 weeks before and 2 weeks after aspiration sclerotherapy to reach optimal drug levels around the procedure. Controls received placebo injections of sodium chloride 0.9 % (Fresenius Kabi, Hesse, Germany) at equal time points: 2 weeks before and 2 weeks after aspiration sclerotherapy. Injections were omitted if safety laboratory margins (fasting blood sugar, alanine aminotransferase, aspartate aminotransferase, bilirubin and amylase) were exceeded [22]. Injections were administered by unblinded healthcare professionals not involved in the design or analysis of this trial.

### Study endpoints

The primary outcome was the mean proportional change (%) in cyst diameter of the treated hepatic cyst from baseline to week 6, as measured by ultrasonography. Secondary outcomes were change in cyst diameter at 14 and 26 weeks; cyst volume reduction at 6, 14 and 26 weeks; recurrence rate (< 20 % reduction) at 6 and 26 weeks; patient-reported outcomes (change in symptoms and health-related quality of life) at 6 and 26 weeks; and rate and severity of adverse events during trial participation.

### Ultrasonography measurement

All ultrasound measurements were performed by the primary blinded operator (TW) using a 3.5 MHz convex transducer (Acuson X150^TM^, Siemens Healthcare, Erlangen, Germany). At baseline, the indicated cyst was visualized in two planes to measure the maximal orthogonal diameters, from which we calculated one mean diameter (Supplementary Fig. 1). In addition, cyst volume was estimated by the ellipsoid volume formula (D1*D2*D3*0.523) [15]. In case of a dominant cyst, the segmental location and surrounding structures (cysts and vessels) were closely documented to secure identification of the treated cyst at follow-up. Measurements were repeated at weeks 6, 14 and 26 to obtain the proportional (%) change. A second blinded independent operator (ML) repeated all measurements of all patients to assess interobserver variability. In addition, the primary operator repeated measurements in a subset of nine patients to evaluate intra-observer variability.

### Patient-reported outcomes

To evaluate symptoms, patients completed the Polycystic Liver Disease-Questionnaire (PLD-Q), which assesses frequency and discomfort of 13 PLD-related symptoms over a timeframe of one month [24]. Scores can be compiled to a total sumscore from 1 (asymptomatic) to 100 (severe symptomatic) points. To evaluate health-related quality of life (HRQL) we used the generic Medical Outcomes Study 36-item short-form health survey (SF-36) [25]. Scores were summarized to a norm-based physical (PCS) and mental component score (MCS), with a general population reference of 50 ± 10 [26]. Lower PCS or MCS scores indicate a compromised HRQL [27].

### Statistical analysis

Based on data from a previous cohort [28], we expected 30 % cyst diameter reduction 4 weeks after aspiration sclerotherapy. We hypothesized that this reduction at 4 weeks would be improved to 50 % by adjuvant treatment with pasireotide. In order to detect a statistical difference between groups, we needed a sample size of 34 participants (power: 80 %; α: 0.05; drop-out rate: 10 %). Intention-to-treat analysis was performed including all patients who received at least one dose of pasireotide. In addition, a parallel per-protocol analysis was conducted in patients who received both injections and aspiration sclerotherapy. Values were reported as median with interquartile range. For continuous endpoints, we calculated differences between arms using the Mann-Whitney U test. To correct for baseline diameter differences and underlying diagnosis (solitary cyst or PLD) between arms, we performed a sensitivity analysis using ANCOVA. The Wilcoxon signed-rank test was used to compare within-group differences. Cyst recurrence was compared between groups by Fisher’s exact test. In case of a missing variable, we carried the last available value forward. Summarizing scores of patient-reported outcomes (PLD-Q sumscore, PCS, MCS) were compared between groups after treatment. To assess safety, we compiled frequency tables for adverse events classified according to WHO adverse reaction terminology. Statistical analyses were performed prior to unblinding using SPSS (version 22.0; SPSS, Chicago, IL, USA). Analyses were two-sided with a *p*-value < 0.05 considered statistically significant.

## Results

### Baseline characteristics

**Fig. 1:**
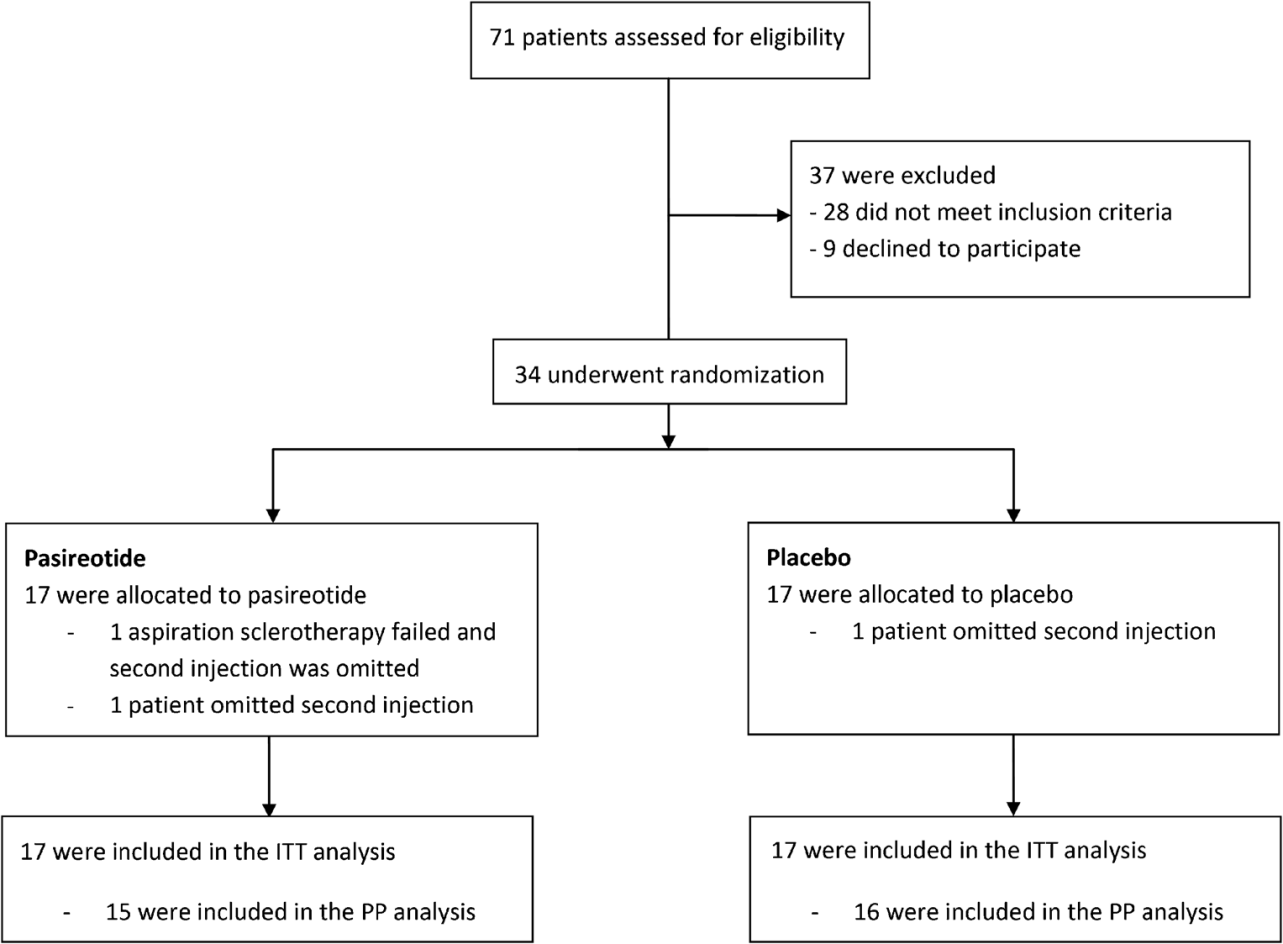
Flow diagram. *ITT* intention-to-treat, *PP* per-protocol

We screened 71 consecutive patients with symptomatic hepatic cysts for eligibility. Of these, 28 patients did not meet the inclusion criteria and nine patients declined to participate (Fig. 1). In total, 34 participants (female n = 32, male n = 2) were randomly assigned to either pasireotide or placebo. Baseline characteristics are shown in Table 1.

**Table 1:** Demographics and baseline characteristics

	Pasireotide (n = 17)	Placebo (n = 17)
Age at treatment, years	54 [51–61]	50 [45–61]
Female sex, n	16 (94)	16 (94)
PLD (>20 cysts), n	10 (59)	13 (76)
Baseline cyst diameter, cm	10.4 [8.2–13.4]	9.0 [7.1–10.8]
Baseline cyst volume, ml	529.8 [288.4–1027.1]	378.2 [188.5–635.7]
BMI, kg/m^2^	27.3 [25.1–30.2]	23.2 [22.2–27.4]

Continuous data are reported as median [interquartile range], absolute numbers as n (%)

*PLD* polycystic liver disease, *BMI* body mass index

All subjects were included in the intention-to-treat analysis. One aspiration sclerotherapy procedure failed as the targeted cyst could not be punctured due to a deep cranial position. This patient did not receive a second injection. In both groups one patient did not receive a second injection due to adverse events (hyperglycaemia and dizziness). Finally, one patient (pasireotide-arm) had a second aspiration sclerotherapy procedure at week 24 due to ongoing severe complaints. From another patient (placebo-arm), the treated cyst could not be identified from other cysts at week 26. Findings at week 14 of these two patients were carried forward to week 26 for analysis.

### Primary outcome

#### Intention-to-treat analysis

**Fig. 2:**
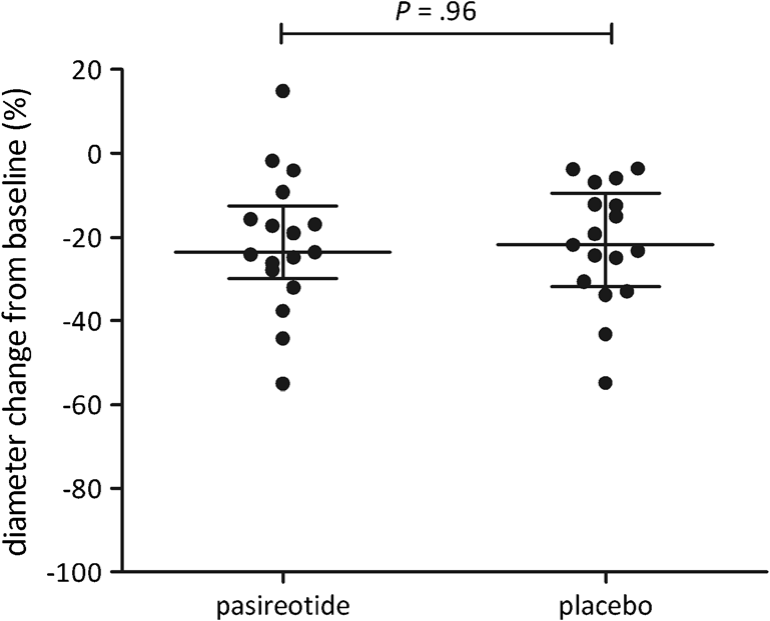
Primary outcome. Diameter reduction compared to baseline at week 6

Median cyst diameter in the pasireotide-arm decreased by 23.6 % (interquartile range [IQR] 12.6–30.0) compared to 21.8 % (IQR 9.6–31.8) in patients assigned to placebo (Fig. 2). Proportional diameter reduction was similar (*p* = 0.96) between groups. When corrected for baseline diameter and diagnosis by ANCOVA, proportional reduction remained equal (*p* = 0.64).

#### Per-protocol analysis

The three patients who did not receive a second injection were excluded from per-protocol analysis, resulting in a sample of 31 patients (Fig. 1). In the pasireotide-sample (n = 15), cyst diameter reduced by 23.6 % (IQR 15.8–32.0) compared to 22.6 % (IQR 12.4–32.4) in patients receiving placebo (n = 16). Similar to the intention-to-treat analysis, we found no statistical significant difference between groups (*p* = 0.83).

#### Intra- and interobserver variability

We found a mean difference of 0.45 % ± 2.72 % between measurements of the primary operator with an intraclass coefficient of r = 0.99 (*p* < 0.01). Compared to measurements of the second operator there was a mean difference of 1.75 % ± 5.30 %, with an intraclass coefficient of r = 0.97 (*p* < 0.01).

### Secondary outcomes

#### Long-term cyst diameter reduction

**Fig. 3:**
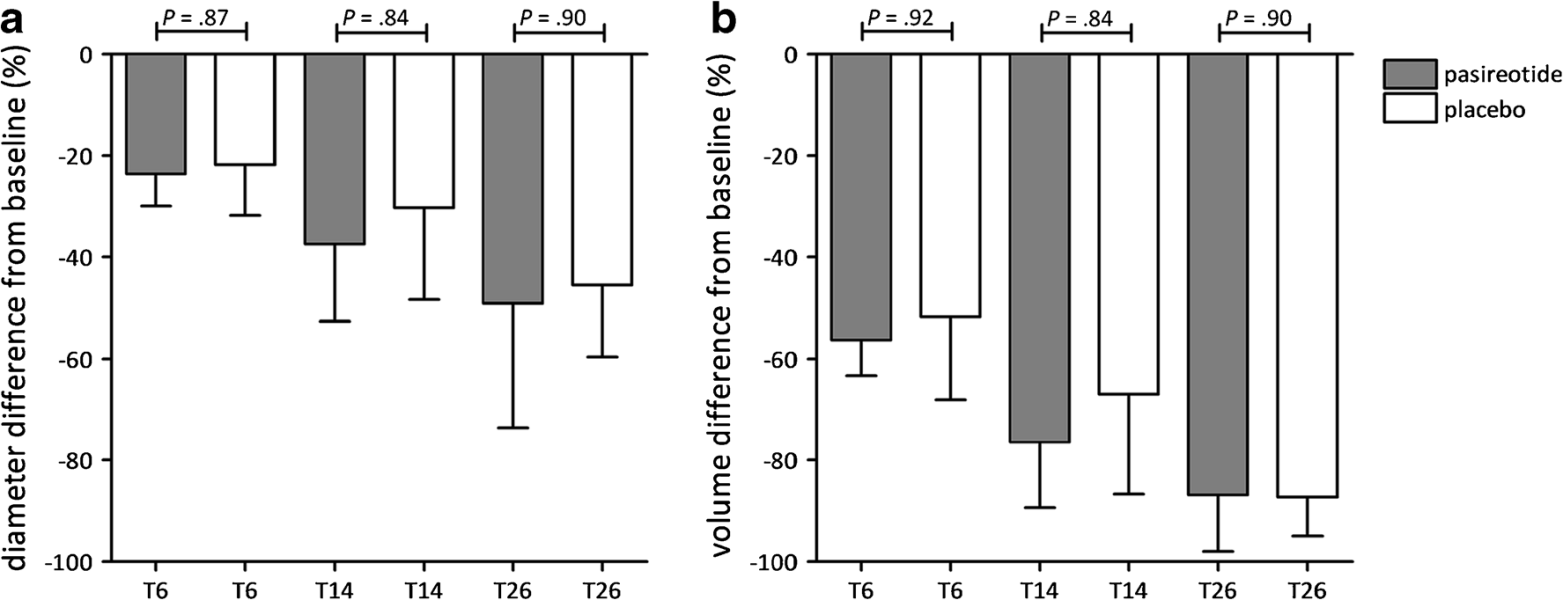
Cyst diameter (**A**) and volume (**B**) reduction from baseline to weeks 6, 14 and 26 compared between pasireotide and placebo

The two groups showed similar progressive long-term diameter reduction (Fig. 3). At week 14, median proportional cyst diameter reduction was 37.5 % (IQR 24.0–52.6) in patients assigned to pasireotide and 30.3 % (IQR 19.5–48.4) in controls (*p* = 0.84). At week 26, treated cyst diameters had further reduced to 49.1 % (IQR 27.0–73.6) (pasireotide) and 45.6 % (IQR 29.6–59.6) (placebo) compared to baseline (*p* = 0.90; Supplementary Table 1).

#### Volume reduction and cyst recurrence

Changes in volume from baseline to 26 weeks are shown in Fig. 3. Similar to diameter results, groups achieved comparable volume reduction rates (Supplementary Table 2). In both groups, 8/17 (47 %) patients showed cyst recurrence at week 6. This decreased to one patient in the pasireotide group and two patients in the placebo group at the end of the trial (*p* = 0.6).

## Patient-reported outcomes

**Fig. 4:**
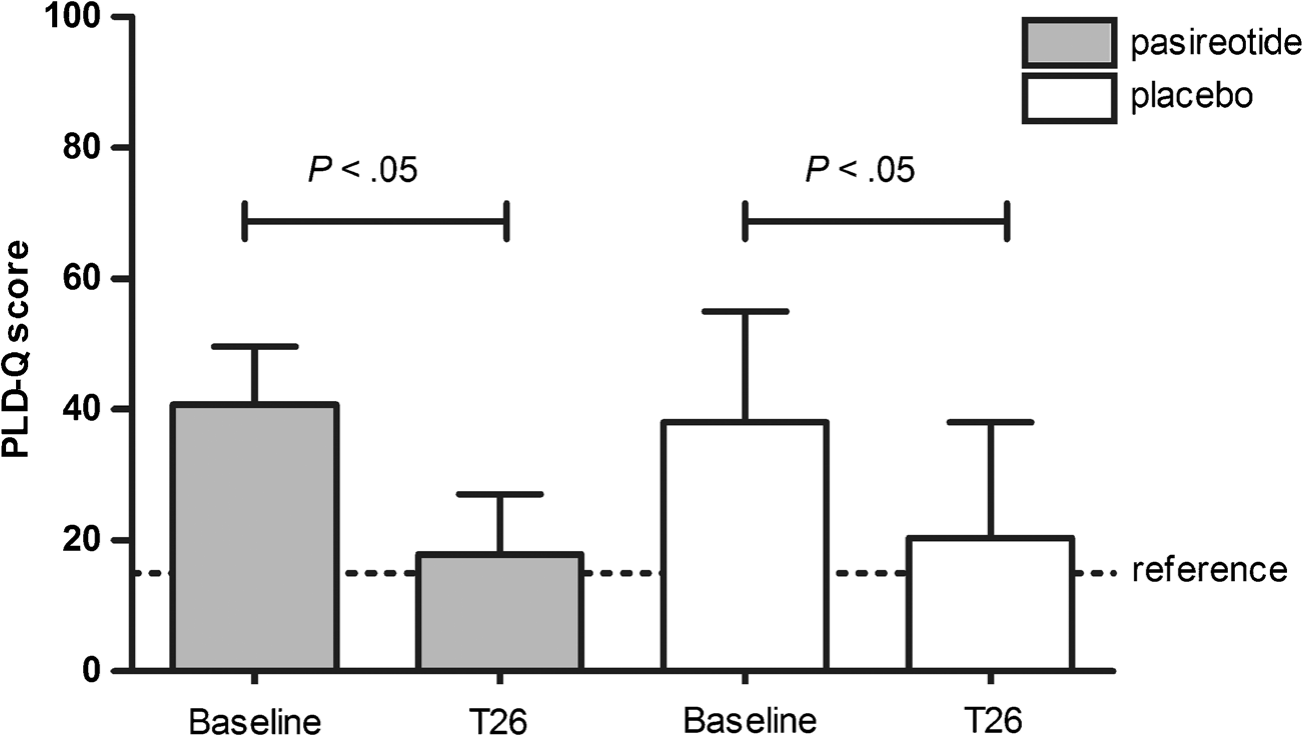
Symptomatic improvement from baseline to week 26 (T26), assessed by the disease-specific Polycystic Liver Disease Questionnaire (PLD-Q) leading to a summarizing score. The dotted line (reference) indicates the general population score

Total PLD-Q score showed a statistically significant (*p* < 0.01) decrease over time in both groups indicating symptomatic improvement (Fig. 4). Final PLD-Q improvement was comparable between groups (*p* = 0.95; Supplementary Table 3). At baseline, the physical domain of HRQL was restricted at baseline in both arms. After treatment, PCS improved in both groups with a statistically significant effect in the pasireotide sample (*p* < 0.01). Nonetheless, PCS at week 26 was similar among arms (*p* = 0.43). Baseline MCS was slightly restricted in the pasireotide group and normal in controls. In both groups the MCS increased; however, the final MCS was similar (*p* = 0.56).

### Adverse events

There were four serious adverse events requiring hospital admission. One female patient developed a cyst-bleeding 10 days after aspiration sclerotherapy that was treated with analgesics. A male patient developed urolithiasis that was treated surgically. Furthermore, a female patient developed pneumonia, which was treated with antibiotics and analgesics. Finally, a female patient underwent a second aspiration sclerotherapy of the treated cyst due to ongoing severe abdominal distension and inadequate cyst reduction from the first procedure.

The most common adverse event was abdominal pain (pasireotide n = 13; placebo n = 11), which was mild and self-limiting in 20 cases (Table 2). In the remaining four cases, pain was more severe (grade III) and analgesics were needed to relieve symptoms. Glucose levels in the pasireotide group rose from 5.3 mmol/L (IQR 5.1–6.1 mmol/L) to 6.8 mmol/L (IQR 6.6–7.7 mmol/L) at week 4 (*p* < 0.01) and normalized at the end of follow-up. By contrast, median levels in patients on placebo did not differ over time (Supplementary Fig. 2).

**Table 2:** Adverse events

	Pasireotide (n = 17)		Placebo (n = 17)	
	Total	Grade 3	Total	Grade 3
Hyperglycaemia	17 (100)	0	5 (29)	0
Abdominal pain	13 (76)	1 (6)	11 (65)	3 (18)
Loose stools	13 (76)	0	10 (58)	0
Fatigue	10 (58)	0	7 (41)	0
Nausea	8 (47)	0	6 (35)	0
Skin response injection site	6 (35)	0	1 (6)	0
Palpitations	4 (24)	0	1 (6)	0
Vomiting	3 (18)	0	1 (6)	0
Decreased satiety	3 (18)	0	1 (6)	0
Overall malaise	3 (18)	0	1 (6)	0
Menstruation disorders^1^	2 (13)	0	0	0
Dizziness	2 (12)	0	2 (12)	0
Thrombotic events^2^	2 (12)	1	0	0
Pneumoniae/pneumonitis	1 (6)	0	1 (6)	1 (6)
Urolithiasis	1 (6)	1 (6)	0	0
Serious adverse events^3^	2 (12)		2 (12)	

Values are given as number (percent)

^1^Prolonged period (n = 1); irregular menstruation (n = 1)

^2^Thrombosis vena subclavia after peripheral catheter left arm (n = 1); superficial thrombosis lower right leg (n = 1)

^3^Serious adverse events: cyst bleeding (n = 1); urolithiasis (n = 1); re-intervention hepatic cyst (n = 1); pneumonia (n = 1)

## Discussion

The key finding of this study is that a pre- and postprocedural intramuscular injection of pasireotide LAR in addition to aspiration sclerotherapy does not improve cyst reduction or clinical outcomes compared to controls. In both arms, we found symptomatic relief and normalized HRQL, underlining the clinical benefit that can be achieved by aspiration sclerotherapy.

We expected that a combined approach to target cysts, i.e. pasireotide and aspiration sclerotherapy, would enhance efficacy over mere aspiration sclerotherapy. This hypothesis was based upon the working mechanism of somatostatin analogues. Somatostatin analogues lower intracellular levels of 3’-5’-cyclic adenosine monophosphate (cAMP) by binding somatostatin receptors (SSTR) [3, 17]. This second messenger cAMP stimulates cyst fluid production by activating chloride (cystic fibrosis transmembrane conductance regulator; CFTR) and water (aquaporin 1) channels on the apical side of the cyst epithelial cells [29]. In addition, cAMP stimulates cell hyperproliferation leading to cyst growth [30, 31]. Therefore, by lowering intracellular levels of cAMP, cyst fluid production is curtailed and epithelial cell proliferation suppressed, collectively leading to cyst regression [17, 31]. Previous clinical studies showed how somatostatin analogues reduce polycystic liver volume [18]. We expected that these features would reduce post-intervention fluid reaccumulation and improve long-term cyst reduction.

Nonetheless, we found no differences between groups. Our efficacy results were comparable with other studies that performed mere aspiration sclerotherapy [7]. Why was our hypothesis not recapitulated by the results of our study? First, while other somatostatin analogues such as lanreotide and octreotide decrease hepatic cyst volumes, it is possible that pasireotide does not inhibit hepatic cystogenesis in humans. Compared to conventional somatostatin analogues, pasireotide has a broader binding profile (octreotide SSTR _2, 3,_ and SSTR_5_; pasireotide SSTR _1,2,3,_ and SSTR_5_) and a higher affinity to SSTR _1, 3_ and SSTR_5_ [20]. Based on this pharmacological profile, it is reasonable to assume that pasireotide also reduces cyst progression in humans. However, no human studies have reported the effects of pasireotide on hepatic cysts. Currently, a randomized clinical trial is being conducted to investigate the efficacy of pasireotide in polycystic liver disease, which will provide better insight into the cyst-reducing properties of pasireotide (Clinicaltrials.gov; NCT01670110).

In addition, we may have administrated pasireotide in an inadequate dosage or regimen to be effective. In order to optimize the efficacy/safety balance we targeted our patients with pasireotide in a limited period before and after the procedure. To reach optimal drug levels, we administered a dosage of 60 mg in a long-acting release formulation. This is the highest available dosage within the safety range [32, 33]. The mild hyperglycaemia that followed pasireotide injections suggests adequate exposure to the drug.

Finally, the process of post-procedural cyst fluid reaccumulation leading to recurrence may not be affected by pasireotide. Alternatively, it is hypothesized that fluid reaccumulation results from an inflammatory response induced by cell lysis following sclerotherapy, rather than fluid production of remaining functional cyst epithelium [13]. An exploratory study repunctured treated cysts 2–8 days following aspiration sclerotherapy and observed an increase in leukocytes compared to the primary aspirate, suggesting ongoing inflammation [34].

Traditionally, efficacy studies of aspiration sclerotherapy focused on the technical (morphological) response of aspiration sclerotherapy [5, 7]. As hepatic cysts are benign lesions, indication for treatment is driven upon the patient’s symptomatology. This was the first study to assess patient-reported outcomes at standardized time points using validated instruments. At baseline, we found low physical SF-36 scores and increased PLD-Q scores indicating a restricted HRQL a symptomatic burden compared to general population references [26]. In this study we demonstrated that aspiration sclerotherapy significantly reduces disease-specific symptoms and normalizes HRQL. These findings support the notion that aspiration sclerotherapy is an excellent first-line choice to treat patients with large symptomatic hepatic cysts.

The strength of this study is that we combined an invasive and medical strategy to optimize cyst reduction in a clinical trial design. This study also has several limitations. Firstly, our primary endpoint was measured by ultrasonography, which introduces variability as it is operator-dependent. CT or MRI may reduce variability, but would have led to unnecessary exposure to radiation and/or costs as this study required multiple measurements within short intervals. To minimize bias, all measurements were performed by the same investigator following standardized operating procedures. As a result, we found excellent intraclass coefficients of intra- and interobserver variability [35]. Secondly, we powered our study assuming a large beneficial effect from pasireotide. Possible smaller differences between our groups cannot be ruled out. However, we believe that such small differences would not be relevant for clinical practice, given the costs of pasireotide. Finally, we included both sporadic hepatic cysts and dominant hepatic cysts within PLD. The genetic background of both congenital cyst types corresponds as somatic loss of PLD-type alleles from cyst epithelium is required to drive cyst formation [36]. Indeed, both cyst types have a thin wall lined by a single layer of aberrant cholangiocytes, which are targetable by somatostatin analogues [4, 37]. However, it is unclear if germline mutations in PLD patients alter susceptibility to these agents. In our study, we found no differences in efficacy between patients with or without underlying PLD, as reflected in the previous literature [8].

In summary, adjuvant pasireotide treatment to aspiration sclerotherapy in patients with large hepatic cysts is no more effective than aspiration sclerotherapy alone. Aspiration sclerotherapy normalizes HRQL-rates and decreases symptomatic disease, which proves aspiration sclerotherapy is an excellent choice of therapy for large hepatic cysts.

## Electronic supplementary material

ESM 1 (DOCX 16.0 kb)

ESM 2 (DOCX 17.4 kb)

ESM 3 (DOCX 18.1 kb)

ESM 4 (DOCX 102 kb)

ESM 5 (DOCX 4.14 kb)

## References

[1] Gevers TJ, Drenth JP (2013). Diagnosis and management of polycystic liver disease. Nat Rev Gastroenterol Hepatol.

[2] Janssen MJ, Waanders E, Te Morsche RH (2011). Secondary, somatic mutations might promote cyst formation in patients with autosomal dominant polycystic liver disease. Gastroenterology.

[3] Perugorria MJ, Masyuk TV, Marin JJ (2014). Polycystic liver diseases: advanced insights into the molecular mechanisms. Nat Rev Gastroenterol Hepatol.

[4] Cowles RA, Mulholland MW (2000). Solitary hepatic cysts. J Am Coll Surg.

[5] Drenth JP, Chrispijn M, Nagorney DM, Kamath PS, Torres VE (2010). Medical and surgical treatment options for polycystic liver disease. Hepatology.

[6] Wijnands TFM, Neijenhuis MK, Kievit W (2014). Evaluating health-related quality of life in patients with polycystic liver disease and determining the impact of symptoms and liver volume. Liver Int.

[7] Wijnands TF, Gortjes AP, Gevers TJ (2017). Efficacy and Safety of Aspiration Sclerotherapy of Simple Hepatic Cysts: A Systematic Review. AJR Am J Roentgenol.

[8] Benzimra J, Ronot M, Fuks D (2014). Hepatic cysts treated with percutaneous ethanol sclerotherapy: time to extend the indications to haemorrhagic cysts and polycystic liver disease. Eur Radiol.

[9] Moorthy K, Mihssin N, Houghton PW (2001). The management of simple hepatic cysts: sclerotherapy or laparoscopic fenestration. Ann R Coll Surg Engl.

[10] Bean WJ, Rodan BA (1985). Hepatic cysts: treatment with alcohol. AJR Am J Roentgenol.

[11] Wills ES, Roepman R, Drenth JP (2014). Polycystic liver disease: ductal plate malformation and the primary cilium. Trends Mol Med.

[12] Okano A, Hajiro K, Takakuwa H, Nishio A (2000). Alcohol sclerotherapy of hepatic cysts: its effect in relation to ethanol concentration. Hepatol Res.

[13] Hahn ST, Han SY, Yun EH (2008). Recurrence after percutaneous ethanol ablation of simple hepatic, renal, and splenic cysts: is it true recurrence requiring an additional treatment?. Acta Radiol.

[14] Wijnands TFM, Ronot M, Gevers TJG et al (2016) Predictors of treatment response following aspiration sclerotherapy of hepatic cysts: an international pooled analysis of individual patient data. Eur Radiol. 10.1007/s00330-016-4363-x:1-8 10.1007/s00330-016-4363-x PMC5209423 27180184

[15] Larssen TB, Rosendahl K, Horn A, Jensen DK, Rorvik J (2003). Single-session alcohol sclerotherapy in symptomatic benign hepatic cysts performed with a time of exposure to alcohol of 10 min: initial results. Eur Radiol.

[16] Tikkakoski T, Makela JT, Leinonen S (1996). Treatment of symptomatic congenital hepatic cysts with single-session percutaneous drainage and ethanol sclerosis: technique and outcome. J Vasc Interv Radiol.

[17] Masyuk TV, Masyuk AI, Torres VE, Harris PC, Larusso NF (2007). Octreotide inhibits hepatic cystogenesis in a rodent model of polycystic liver disease by reducing cholangiocyte adenosine 3',5'-cyclic monophosphate. Gastroenterology.

[18] Gevers TJ, Inthout J, Caroli A (2013). Young women with polycystic liver disease respond best to somatostatin analogues: a pooled analysis of individual patient data. Gastroenterology.

[19] Chrispijn M, Nevens F, Gevers TJ (2012). The long-term outcome of patients with polycystic liver disease treated with lanreotide. Aliment Pharmacol Ther.

[20] Schmid HA (2008). Pasireotide (SOM230): development, mechanism of action and potential applications. Mol Cell Endocrinol.

[21] Vauthey JN, Maddern GJ, Kolbinger P, Baer HU, Blumgart LH (1992). Clinical experience with adult polycystic liver disease. Br J Surg.

[22] Wijnands TF, Gevers TJ, Kool LJ, Drenth JP (2015). Aspiration sclerotherapy combined with pasireotide to improve reduction of large symptomatic hepatic cysts (SCLEROCYST): study protocol for a randomized controlled trial. Trials.

[23] van Keimpema L, de Koning DB, Strijk SP, Drenth JP (2008). Aspiration-sclerotherapy results in effective control of liver volume in patients with liver cysts. Dig Dis Sci.

[24] Neijenhuis MK, Gevers TJ, Hogan MC et al (2016) Development and validation of a disease-specific questionnaire to assess patient-reported symptoms in polycystic liver disease. Hepatology. 10.1002/hep.28545 10.1002/hep.28545 PMC4917464 26970415

[25] Ware JE (1993). SF-36 health survey : manual and interpretation guide.

[26] Ware JE, Gandek B, Kosinski M (1998). The equivalence of SF-36 summary health scores estimated using standard and country-specific algorithms in 10 countries: results from the IQOLA Project. International Quality of Life Assessment. J Clin Epidemiol.

[27] Ware JE, Kosinski M, Keller SD (1994). SF-36 physical and mental health summary scales : a user's manual.

[28] Chrispijn M, Weimer FH, El-Massoudi Y (2012). Treatment Success of Aspiration and Sclerotherapy for Hepatic Cysts depends on Cyst Diameter and Volume of Sclerosing Agent. Hepatology.

[29] Banales JM, Masyuk TV, Bogert PS (2008). Hepatic cystogenesis is associated with abnormal expression and location of ion transporters and water channels in an animal model of autosomal recessive polycystic kidney disease. Am J Pathol.

[30] Masyuk TV, Radtke BN, Stroope AJ (2013). Pasireotide is more effective than octreotide in reducing hepatorenal cystogenesis in rodents with polycystic kidney and liver diseases. Hepatology.

[31] Masyuk TV, Hogan MC, LaRusso NF (2016) Polycystic Liver Disease: The benefits of targeting cAMP. Clin Gastroenterol Hepatol. 10.1016/j.cgh.2016.03.008 10.1016/j.cgh.2016.03.008 PMC4912886 26972981

[32] Dietrich H, Hu K, Ruffin M (2012). Safety, tolerability, and pharmacokinetics of a single dose of pasireotide long-acting release in healthy volunteers: a single-center Phase I study. Eur J Endocrinol.

[33] Petersenn S, Bollerslev J, Arafat AM (2014). Pharmacokinetics, pharmacodynamics, and safety of pasireotide LAR in patients with acromegaly: a randomized, multicenter, open-label, phase I study. J Clin Pharmacol.

[34] Larssen TB, Rorvik J, Horn A (2004). Biochemical and cytologic analysis of cystic contents in benign non-parasitic symptomatic hepatic cysts before and after ethanol sclerotherapy. Acta Radiol.

[35] Koo TK, Li MY (2016). A Guideline of Selecting and Reporting Intraclass Correlation Coefficients for Reliability Research. J Chiropr Med.

[36] Janssen MJ, Salomon J, Cnossen WR, Bergmann C, Pfundt R, Drenth JP (2015). Somatic loss of polycystic disease genes contributes to the formation of isolated and polycystic liver cysts. Gut.

[37] Wills ES, Roepman R, Drenth JPH (2014). Polycystic liver disease: ductal plate malformation and the primary cilium. Trends Mol Med.

