# Supplementary material for: Pasireotide does not improve efficacy of aspiration sclerotherapy in patients with large hepatic cysts, a randomized controlled trial
Source: Eur Radiol. 2018 Jan 9;28(6):2682–9. doi: 10.1007/s00330-017-5205-1 (PMC5938297; doi:10.1007/s00330-017-5205-1)
Supplement: Supplementary file 1 — (DOCX 16.0 kb) [file 330_2017_5205_MOESM1_ESM.docx]

**SUPPLEMENTARY TABLES**

**Supplementary Table 1. Median proportional (%) diameter reduction compared to baseline**

|  | **Pasireotide (n = 17)** | **Placebo (n = 17)** | ***P* value** |
| --- | --- | --- | --- |
| Week 6, % | 23.6 [12.6-30.0] | 21.8 [9.6-31.8] | 0.865 |
| Week 14, % | 37.5 [24.0-52.6] | 30.3 [19.5-48.4] | 0.838 |
| Week 26, % | 49.1 [27.0-73.6]^1^ | 45.6 [29.6-59.6]^1^ | 0.901 |

Data are reported in median [interquartile range] and analysed by Mann Whitney U test; ^1^ last value carried forward in pasireotide-arm (n = 1) and placebo-arm (n = 1).
