# Supplementary material for: Pasireotide does not improve efficacy of aspiration sclerotherapy in patients with large hepatic cysts, a randomized controlled trial
Source: Eur Radiol. 2018 Jan 9;28(6):2682–9. doi: 10.1007/s00330-017-5205-1 (PMC5938297; doi:10.1007/s00330-017-5205-1)
Supplement: Supplementary file 2 — (DOCX 17.4 kb) [file 330_2017_5205_MOESM2_ESM.docx]

**SUPPLEMENTARY TABLES**

**Supplementary Table 2. Volume proportional (%) reduction compared to baseline**

|  | **Pasireotide (n = 17)** | **Placebo (n = 17)** | ***P* value** |
| --- | --- | --- | --- |
| Week 6, % | 56.3 [31.0-63.3] | 51.8 [25.0-68.0] | 0.919 |
| Week 14, % | 76.4 [55.5-89.3] | 67.0 [46.8-86.7] | 0.838 |
| Week 26, % | 86.9 [60.0-98.0]^1^ | 83.1 [66.3-93.4]^1^ | 0.901 |

Data are reported in median [interquartile range] and analysed by Mann Whitney U test; ^1^ last value carried forward in pasireotide-arm (n = 1) and placebo-arm (n = 1).
