# Supplementary material for: Pasireotide does not improve efficacy of aspiration sclerotherapy in patients with large hepatic cysts, a randomized controlled trial
Source: Eur Radiol. 2018 Jan 9;28(6):2682–9. doi: 10.1007/s00330-017-5205-1 (PMC5938297; doi:10.1007/s00330-017-5205-1)
Supplement: Supplementary file 3 — (DOCX 18.1 kb) [file 330_2017_5205_MOESM3_ESM.docx]

**SUPPLEMENTARY TABLES**

**Supplementary Table 3 - Patient-reported outcome measures**

|  | **Pasireotide (n = 17)** | |  | **Placebo (n = 17)** | | |
| --- | --- | --- | --- | --- | --- | --- |
|  | Baseline | Week 6 | Week 26 | Baseline | Week 6 | Week 26 |
| **PLD-Q** | 40.7 [26.3-49.7] | 25.9 [22.2-37.2]* | 19.9 [12.5-27.1]* | 38.0 [26.3-55.0] | 29.1 [15.0-49.6]* | 20.4 [11.1-38.0]* |
| **SF-36**  **- PCS** | 39.4 [35.1-51.2] | 46.1 [40.1-52.1] | 51.9 [41.1-56.3]* | 43.0 [30.0-53.4] | 48.5 [31.1-52.9] | 49.7 [40.4-53.8] |
| **- MCS** | 48.0 [41.8-56.5] | 53.5 [41.3-57.4] | 55.9 [48.0-57.9]* | 53.9 [46.0-61.1] | 52.7 [43.3-59.7] | 56.8 [49.2-60.1] |

Variables are shown as median [interquartile range]; * (P < .05) compared to baseline using Wilcoxon signed-rank test; final scores were similar between arms. PLD-Q = Polycystic Liver Disease-Questionnaire; SF-36 = Medical Outcomes Study 36-item short-form health survey; PCS = physical component score; MCS = mental component score
