# Supplementary material for: Pasireotide does not improve efficacy of aspiration sclerotherapy in patients with large hepatic cysts, a randomized controlled trial
Source: Eur Radiol. 2018 Jan 9;28(6):2682–9. doi: 10.1007/s00330-017-5205-1 (PMC5938297; doi:10.1007/s00330-017-5205-1)
Supplement: Supplementary file 4 — (DOCX 102 kb) [file 330_2017_5205_MOESM4_ESM.docx]

**SUPPLEMENTARY FIGURE LEGENDS**

**
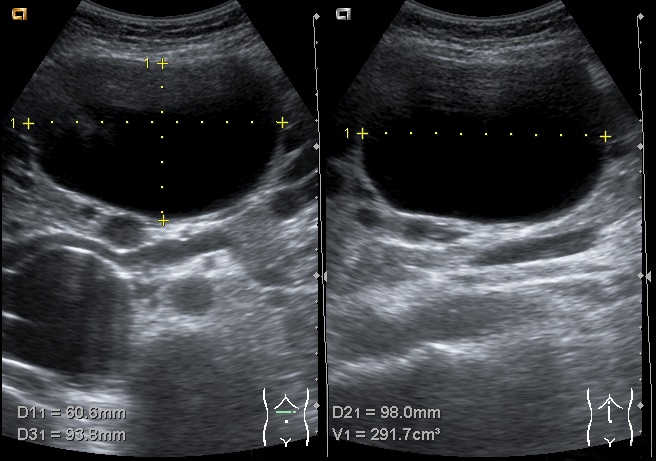
**

**Supplementary Figure 1**: Cyst measurement of orthogonal diameters (anteroposterior, mediolateral, and craniocaudal) in two planes. In this case, a mean diameter of 84.1 mm and volume of 291.7 mL were obtained
