# Supplementary material for: Pasireotide does not improve efficacy of aspiration sclerotherapy in patients with large hepatic cysts, a randomized controlled trial
Source: Eur Radiol. 2018 Jan 9;28(6):2682–9. doi: 10.1007/s00330-017-5205-1 (PMC5938297; doi:10.1007/s00330-017-5205-1)
Supplement: Supplementary file 5 — (DOCX 4.14 kb) [file 330_2017_5205_MOESM5_ESM.docx]

**SUPPLEMENTARY FIGURE LEGENDS**


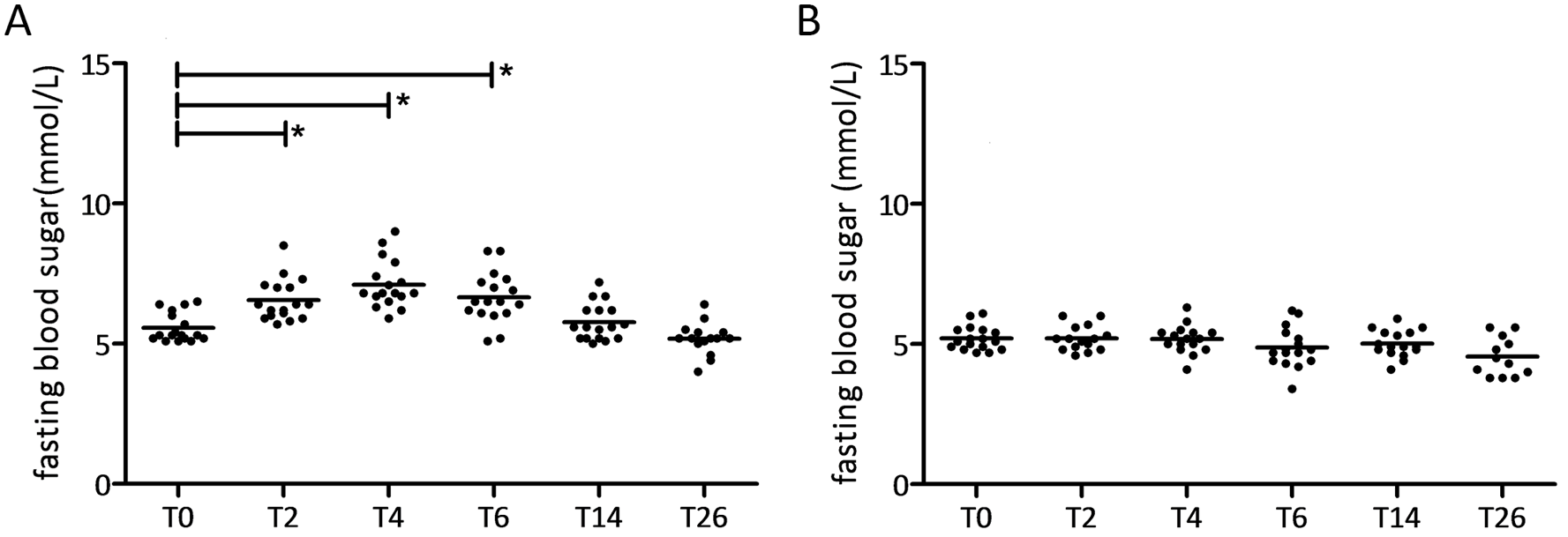


**Supplementary Figure 2:** Fasting blood sugar levels in pasireotide (A) and placebo (B) arms, * = P < 0.0001
